# Technologies and Formulation Design of Polysaccharide-Based Hydrogels for Drug Delivery

**DOI:** 10.3390/molecules25143156

**Published:** 2020-07-10

**Authors:** Giulia Auriemma, Paola Russo, Pasquale Del Gaudio, Carlos A. García-González, Mariana Landín, Rita Patrizia Aquino

**Affiliations:** 1 Department of Pharmacy, University of Salerno, Via Giovanni Paolo II 132, I—84084 Fisciano (SA), Italy; gauriemma@unisa.it (G.A.); paorusso@unisa.it (P.R.); pdelgaudio@unisa.it (P.D.G.); 2 Department of Pharmacy and Pharmaceutical Technology, University of Santiago de Compostela, 15782 Santiago de Compostela, Spain; carlos.garcia@usc.es (C.A.G.-G.); m.landin@usc.es (M.L.)

**Keywords:** polysaccharides, hydrogels, prilling, droplets, ionotropic gelation, drying, xerogels, cryogels, aerogels

## Abstract

Polysaccharide-based hydrogel particles (PbHPs) are very promising carriers aiming to control and target the release of drugs with different physico-chemical properties. Such delivery systems can offer benefits through the proper encapsulation of many drugs (non-steroidal and steroidal anti-inflammatory drugs, antibiotics, etc) ensuring their proper release and targeting. This review discusses the different phases involved in the production of PbHPs in pharmaceutical technology, such as droplet formation (SOL phase), sol-gel transition of the droplets (GEL phase) and drying, as well as the different methods available for droplet production with a special focus on prilling technique. In addition, an overview of the various droplet gelation methods with particular emphasis on ionic cross-linking of several polysaccharides enabling the formation of particles with inner highly porous network or nanofibrillar structure is given. Moreover, a detailed survey of the different inner texture, in xerogels, cryogels or aerogels, each with specific arrangement and properties, which can be obtained with different drying methods, is presented. Various case studies are reported to highlight the most appropriate application of such systems in pharmaceutical field. We also describe the challenges to be faced for the breakthrough towards clinic studies and, finally, the market, focusing on the useful approach of safety-by-design (SbD).

## 1. Introduction

In the last three decades, there has been a constant development of polysaccharide-based hydrogel particles (PbHPs) as smart tools to release drugs with the right kinetic and target. The encapsulation of an active pharmaceutical ingredient (API) inside these polymeric micro-particles

- gives the possibility to realize controlled release according to specific therapeutic needs
- ensures the protection against the action of environmental and physiological agents
- can modify pharmacokinetic and bio-distribution profiles
- can reduce clearance and side effects
- improve drug targeting.

**Figure 1:**
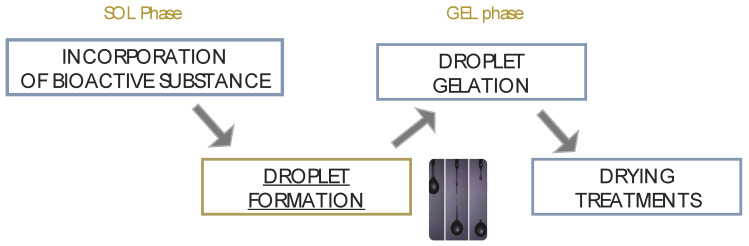
Illustration of the general way for producing hydrogels in form of particles: transition from polysaccharide solution (SOL phase) to a gel particle (GEL phase) followed by possible drying treatments.

Several techniques can be used for the preparation of PbHPs. Many methods are based on the preparation of spherical droplets made by mixtures of the API and the polymeric excipients. For the development of such PbHPs is crucial the polysaccharide droplet formation phase (Figure 1) that in turn defines the size and the size distribution of the resulting microparticles, the two primary factors affecting drug release [1,2,3].

In general, the processes used to prepare monodispersed particles starting from polysaccharide-droplets can be divided into:

(1) Formation of droplets in a gaseous phase with following fall in a gelling medium.
(2) Formation of droplets in a liquid phase that is immiscible with the polymeric solution; in this case, the mixing leads to an emulsion.

For both methodologies, the critical parameters able to determine size and shape of the liquid droplets, are the following: the viscosity of each phase, the surface tension of the polysaccharide solution compared to the surrounding medium (gas/air or liquid) and the dynamic interactions of the droplets with the matrix fluid (laminar or turbulent flow). In case (a), where the liquid is pushed through a nozzle at a constant flow rate, surface tension between droplet and air (liquid-air interface) is essential. In the second case (b), the liquid is broken down in an immiscible fluid system in form of droplets and the interfacial tension between dispersed and continuous phases is usually controlled by surfactants [4].

**Figure 2:**
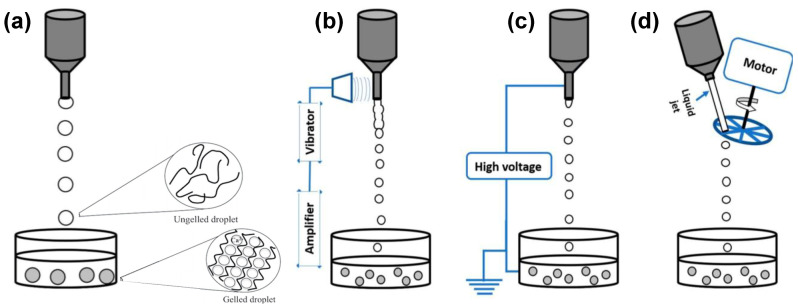
Illustration of dripping devices: (**a**) conventional dripping method influenced by gravity, surface tension and viscosity; breaking up of liquid jets into droplets stimulated by (**b**) vibrating nozzle method, (**c**) electrostatic forces and (**d**) a mechanical cutting device. Reprinted (with some modifications) from [4]. Copyright (2018) Ganesan, Budtova, Ratke, Gurikov, Baudron, Preibisch, Niemeyer, Smirnova, Milow.

As shown in Figure 2, the main processes involving droplet formation in gaseous phase can be grouped according to the mechanism of liquid jet break-up in: simple extrusion (conventional dripping, Figure 2a), vibrating nozzle (Figure 2b), electrostatic (Figure 2c) and mechanical cutting method (Figure 2d).

Conventional dripping has been widely used to produce mainly alginate particles able to encapsulate cells, enzymes, probiotics, plant extracts, oils and flavours [5,6,7,8,9,10,11,12,13,14]. This method involves the manual extrusion of polymeric droplets from a fluid filled syringe or pipette into a gelation or coagulation bath (Figure 2a). When the polysaccharide solution flows out, a droplet is formed at the orifice. The polymeric droplet grows in size until it detaches from the orifice under the influence of gravity, falling toward the gelling medium. In this method, there is no precise control on the formation of the droplets that, during the falling, have the tendency to become spherical due to the surface tension of the liquid before being gelified. Although extrusion by syringe or pipette is the simplest way to produce polysaccharide gel particles, this method generally leads to large gel particles that are polydisperse and not always spherical in shape [4,15,16]. This happens because gravity is the main driving force to generate the droplet from the orifice. In addition, several scale-up difficulties limit this method to a lab scale setup [4,17,18]. Another limitation is represented by the possibility to process only low viscosity feed solutions due to pumping problems and needle blockage [19].

Considering the other technologies illustrated in Figure 2b–d, the breaking up of the polysaccharide liquid jet into droplets is determined by specific devices that give the possibility to strictly control droplet formation [4]. Among them, vibrating nozzle method, also known as prilling or laminar jet break-up, has been widely reported in literature for its great versatility, reproducibility and high scalability potential [20,21,22,23].

The present review surveys the main results gained in prilling technology addressing: (i) the basic aspects of the droplet formation technique, its possible implementations and the ionic crosslinking as main gelation method of the droplets formed by prilling, (ii) the main polysaccharides suitable for PbHPs production by prilling; (iii) the possible approaches exploitable for the ionic gelation, e.g. external, internal or inverse, (iv) the influence of the applied drying method on polymer matrix characteristics and hence on its properties affecting the release of the entrapped drug.

## 2. Prilling Technique to Produce Polymeric Droplets

### Prilling or Laminar Jet Break-Up

Prilling process is based on the mechanical dispersion of the feed solution through pressure-controlled injection in a specific gelation or coagulation medium after breaking apart into mono-sized drops by means of a vibrating nozzle device [23,24]. The technology has been shown especially suitable to immobilize microorganisms or entrap bioactive substances in polymeric beads; these are formed by fall of a mixed host-polymer liquid formulation into an appropriate polymer gelling solution [25,26,27,28]. Recently, many pharmaceutical applications of such beads have been developed in order to control the drug release in orally administered formulations [29,30] or the colon targeting [31,32].

**Figure 3:**
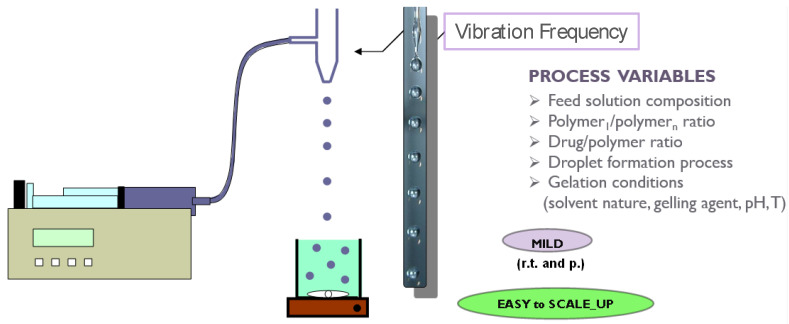
Schematic illustration of prilling technology with the indication of the main process variables.

In the vibrating nozzle method (Figure 3), the monodispersed droplets are formed from a laminar liquid jet by applying superimposed vibrations with an optimal frequency either on the nozzle or on the liquid that is approaching the nozzle. The vibrations can be generated using sound waves (ultrasound) [4,33]; the acoustic jet excitation process involved in prilling was patented to produce uniform microspheres of alginate [34], collagen [35] and PLGA [36]. Practically, polymeric feed solution is pressurized using a pump or gas through a nozzle in order to generate the liquid jet. The superimposed vibrations destabilize the liquid jet (Rayleigh instability) and the jet is disintegrated into monodispersed liquid droplets [3].

Several variables, such as density, dynamic viscosity and flow rate of the feed solution, nozzle geometry and diameter, frequency of vibration as well as falling distance, can affect shape, size and size distribution of the droplets and consequently of the resulting hydrogel particles [16,37,38,39,40].

Viscosity is certainly one of the most important variable of this technique; prilling is able to process only solutions with viscosity values lower than few hundreds of mPa⋅s [21] and it is essential to study the so-called nozzle viscosity (dynamic viscosity) using appropriate theoretical model [23].

As regards to the droplet size, it is estimated to be at least twice the nozzle inner diameter and can be varied by changing the flow rate of the liquid and nozzle diameter [40]. In addition, particle sphericity can be highly influenced by the distance between the vibrating nozzle and the gelling bath. In fact, when the droplets hit the surface of the gelation medium, their spherical shape can be deformed if the droplet viscosity and surface tension forces are unable to overcome the surface tension exerted by the gelling solution [17,41]. Different papers have demonstrated that the liquid droplets are generally able to overcome the impact forming spherical gel particles when the falling distance is greater than 10 cm [15,16]. Uniformity of the polymeric gel particles can also be improved by reducing the surface tension of the gelling bath by the addition of surfactants [15,42]. Moreover, smaller nozzle diameters and higher frequencies increase the possibility of coalescence [24]. For this reason, frequency is usually kept as low as possible in order to avoid the formation of satellite droplets leading to a broader size distribution [29].

**Figure 4:**
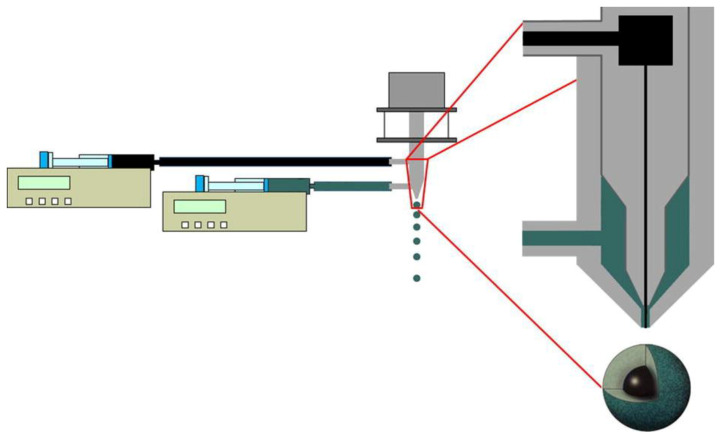
Schematic reproduction of prilling process in co-axial configuration. Reprinted from [43] with permission from Elsevier. Copyright (2014).

Prilling technology can be also used in the co-axial configuration (Figure 4) to obtain droplets with multiple layers formed by different polysaccharides, in a single manufacturing step [43,44]. Core-shell beads can be easily fabricated by prilling in co-axial configuration designing formulations able to obtain a drug controlled release and to diminish the effect of the GI environment. The appropriate combination of two or more polysaccharides may be, for example, an effective way to produce a polymer-drug core (e.g., pectin) enveloped by gastroresistant shell (e.g., alginate) able to prevent the early release of the drug in the upper part of the gastro-intestinal tract (GIT) [45,46,47,48]. An enteric shell may release the drug in a specific district of the organism (intestinal/colonic tract); moreover, from the use of bioadhesive polymers may increase the gastric retention time and, therefore, improve the localized action in GI tract or even delay the release in a precise moment of the day as required, for example, for the treatment of severe chronic mucosal inflammations such as Inflammatory bowel disease IBD [31].

**Figure 5:**
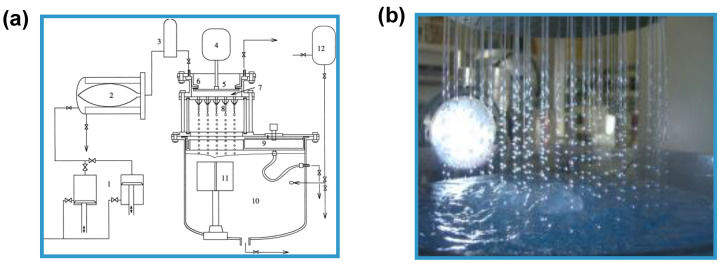
(**a**) Schematic illustration representing a multi-nozzle encapsulator (1, double piston pump; 2, sterile barrier; 3, damper; 4, vibrator; 5, membrane of pulsation chamber; 6, concentric split; 7, pulsation chamber; 8, nozzle plate; 9, bypass system; 10, reaction vessel; 11, stirrer; and 12, input hardening solution). Reprinted from [26] with permission from Elsevier. Copyright (1998). (**b**) Picture showing the equipment supplied by Brace GmbH (https://www.brace.de).

Particle manufacturing through the vibrating nozzle device is easily to scale up e.g., by using a multi-nozzle system (Figure 5) without changing other process parameters such as flow rate and the vibration frequency [21,26]. The most important element is about the arrangement of the nozzles which must ensure equal jet formation and equal pressure drops between the nozzles [26]. The pilot apparatus using this technique is now being sold by some companies such as Brace GmbH (Karlstein am Main, Bavaria, Germany), Nisco Inc. (Zurich, Switzerland), EncapBioSystems AG (Greifensee, Switzerland) [24,49].

Once the droplets are formed, the SOL-GEL transition in hydrogels (gel network formation) must take place as soon as possible to prevent either the aggregation of polymer droplets or the undesired leakage of encapsulated drugs. The chemical nature of the droplets (dispersed phase) determines the subsequent consolidation step, in which the droplets are transformed into solid particles known as gel-beads, involving: (i) non-solvent induced phase separation (NIPS), (ii) temperature or pH modifications, (iii) chemical reactions or ionic cross-linking for water soluble polymers or solvent evaporation/extraction for oil soluble polymers [24]. During the hardening process, the droplets can shrink. The shrinkage is influenced by the type of polysaccharide, its concentration and nature of the hardening medium.

## 3. Methods for the Gelation of Polymeric Droplets to Produce Gel-Particles

**Figure 6:**
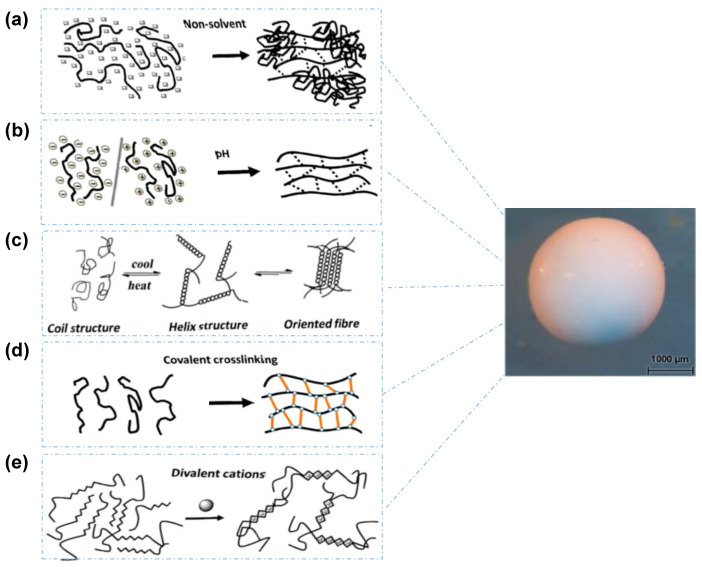
Illustration of the main mechanisms to induce SOL-GEL transition of polysaccharide droplets: (**a**) non-solvent approach to produce a non-solvent filled gel network, (**b**) pH-induced gelation, (**c**) temperature-induced (thermotropic) gelation in which the polysaccharides undergo structural transition from coil to helix and then to double helix, (**d**) covalent crosslinking approach in which the polysaccharide chains are covalently crosslinked to form gel network and (**e**) ions-induced (ionotropic) gelation in which the polysaccharide molecules are crosslinked by ions. Reprinted (with some modifications) from [4]. Copyright (2018) Ganesan, Budtova, Ratke, Gurikov, Baudron, Preibisch, Niemeyer, Smirnova, Milow.

Figure 6 shows the main mechanisms involved in the gelation of polysaccharide droplets to produce gel particles.

### 3.1. Non-Solvent Induced Phase Separation

Non-solvent induced phase separation (NIPS) is also known as coagulation or immersion precipitation. In this case, the polymer is dissolved in a specific solvent and when this solution is extruded into the coagulation bath containing the non-solvent, there is a rapid decrease of polymer solubility leading to phase separation. Polymer chains self-associate and form a 3D self-standing network with the non-solvent in the pores (see Figure 6a). Generally, polysaccharide macromolecules shrink upon the addition of non-solvent, but not completely collapse if polymer concentration is above the overlap concentration. The NIPS process has been applied from several authors to a diverse set of polysaccharides, such as cellulose [50,51], alginate [52,53], pectin [54] and chitin [55]. In these publications, different liquids were exploited as non-solvents to induce phase separation. Pérez-Madrigal et al. studied the ability of aqueous sodium alginates to gelify upon mixing with dimethyl sulfoxide (DMSO) and other organic solvents such as dimethylformamide, methanol, ethanol etc. Gel formation was shown to depend on nature of the non-solvent, solution viscosity (which is correlate to polymer molecular weight and concentration) and gelation time. Similar results were obtained by Tkalec et al. [56,57]. Chitin and chitin-graft-poly(4-vinyl pyridine) were coagulated in ethanol [58,59]; in this case the gelation occurs for the increase of the hydrophobic interactions between the polysaccharide chains, an effect depending on the polymer type. The obtained gel particles are usually defined “alcogels”, an attractive opportunity for aerogels processing by supercritical drying [57,60] as we will discuss in the next paragraphs.

The non-solvent properties of ethanol have been also utilized for hardening of alginate and pectin hydrogel microparticles prepared with other techniques such as emulsion gelation [61,62] and ionic cross-linking [61,62,63,64] with the aim to further stabilize the polymeric gel network by a combination of hydrogen bonds and hydrophobic interactions [53].

### 3.2. pH-Induced Gelation

The pH-induced gelation can be promoted changing pH value of some polysaccharide solvent; at the contact point of each droplet of polymeric solution with the acidic or alkaline bath, the gelation starts forming first a shell, and becomes later complete thanks to the diffusion of the ions through the shell (see Figure 6b). This method is often used alone or in combination with other gelation methods to prepare gel particles of chitosan, pectin and alginic acid [15]. For example, alginic acid gels are formed when pH of the solution is brought down below the disassociation constant (pKa) of the polymer [65]. As reported by Draget et al. [66] also the rate of decrease in pH can affect gel properties; in fact, a rapid decrease in pH results in precipitation of alginic molecules in the form of aggregates while a slow and steady drop in pH results in the formation of a continuous alginic acid bulk gel. Unlike ionic gels, acid gels of alginate are stabilized by intermolecular hydrogen bonds between carboxylic groups of different chains and M-blocks residues have been shown to play a part in gelation. This also applies to pectin, for which the gelation is stabilized by hydrophobic interactions of methylated groups [67,68]. By contrast, chitosan gel particles are prepared at higher pH values. Chitosan is firstly dissolved under mild acidic condition (usually realized using acetic acid) by protonating the amine functional group, and then gel particles are produced under alkaline medium (usually with NaOH solution); the pH value of the alkaline solution must be maintained above the pKa value (6.3) of -NH_2_ functional groups in order to deprotonate the amine groups [4]. Cellulose is instead coagulated using strong acidic solutions of H_2_SO_4_ [69], HNO_3_ [70] or HCl [71,72,73] that, acting as non-solvents, induce the formation of a gel-like structure [4].

### 3.3. Temperature-Induced Gelation

Temperature-induced gelation is also called thermotropic- or cryo-gelation. In this case, the polysaccharide molecules associate themselves often into oriented form, e.g., from coil to helix and then to double helix, in response to temperature, usually upon cooling. The association of these helices leads to double helix formation, then proceeding to a gel network (Figure 6c). Temperature-induced gelation as well as gel properties are highly depending upon polysaccharide typology. In fact, different mechanisms are discussed in the literature, e.g., for agar [74,75,76], κ-carrageenan [76,77], starch [78,79], cellulose [80,81,82] and chitosan [83].

### 3.4. Chemical Gelation

Chemical gelation can be mediated by ionotropic or covalent crosslinking. In the first case, the polysaccharides are crosslinked by ions forming a gel network (Figure 6e). In the second case, gels are formed via covalent cross-linking which leads to irreversible chemical networks (Figure 6d). The main problem is that the majority of covalent cross-linking agents are not biocompatible [4,84]. Among them, glutaraldehyde is certainly that with the longest history; it has been widely used to cross-link several biopolymers such as chitosan [85,86], sodium alginate [87,88,89], cellulose [90,91], guar gum [92,93], collagen [94], collagen-chitosan [95], alginate-guar gum [96], and carrageenan [97,98]. For instance, chitosan microspheres can be produced by mixing chitosan and glutaraldehyde solutions in oil containing surfactants [99,100]. In this case, a Schiff base reaction between amine and aldehyde occurs and as a result, chitosan chains are covalently cross-linked by the glutaraldehyde molecules. With the same mechanism, other aldehydes such as glyoxal and formaldehyde are able to crosslink chitosan chains [101,102,103]. Glutaraldehyde has been highly used also for alginate reticulation. Other covalent gelling agents used for this polymer are adipic dihydrazide, lysine, and poly(ethylene glycol)-diamines [104]. Clearly, the type of cross-linking molecule and the cross-linking density determines both the mechanical properties and the degree of swelling in alginate hydrogels. Usually, cellulose is chemically cross-linked in aqueous solution by using epichlorohydrin, dichlorohydroxytriazine, 1,3,5-triacryloylhexahydrotriazine, 2,4-diacrylamido-benzenesulphonic acid, *N*-methylol resins or dialdehydes [4].

As confirmed by the high number of papers present in the literature, among all gelation techniques, ionotropic cross-linking of polysaccharide solutions is the most investigated for the fabrication of biocompatible systems used in biomedical field, due to its affordability, versatility and high reproducibility [105,106].

### 3.5. Ionotropic Cross-Linking

Ionotropic gelation exploits the capability of polysaccharide-based polyelectrolytes to crosslink in the presence of counter ions under specific ranges of concentration and/or pH [107]. The ionic cross-linking of polysaccharide droplets in aqueous solution gives rise to hydrogel particles (or beads) characterized by a microstructure with interconnected nanofibrillar network [108,109,110,111]. Hydrogel physicochemical properties depend upon chemical composition of the selected polysaccharide, its concentration as well as the size (i.e., ionic radius) and the valence (i.e., coordination number) of counter ions and eventually, the presence of water of hydration surrounding cross-linking ions [2,112].

Biocompatible and biodegradable alginate, pectin, chitosan are polyelectrolytes having active functional groups, such as carboxylate, sulphate and amine that can be involved in the ionotropic gelation mechanism [113]. Obviously, the type of counter ion and the gelling conditions must be chosen in relation to the specific polysaccharide used for droplet formation. Alginate and pectin, being polyanionic polysaccharides, tend to cross-link in presence of polyvalent cations. In this case, the gelation is induced by the electrostatic interactions establishing between cations and the polymer anion blocks [114]. On the contrary, chitosan with its amine functional group can undergo ionotropic gelation in presence of anionic counter ions such as tripolyphosphate (TPP), sulphate and citrate. The gel network formation is due to the electrostatic interactions established between anionic counter ions and the chitosan cationic blocks [115,116,117].

#### 3.5.1. Alginate Ionic Cross-Linking

**Figure 7:**
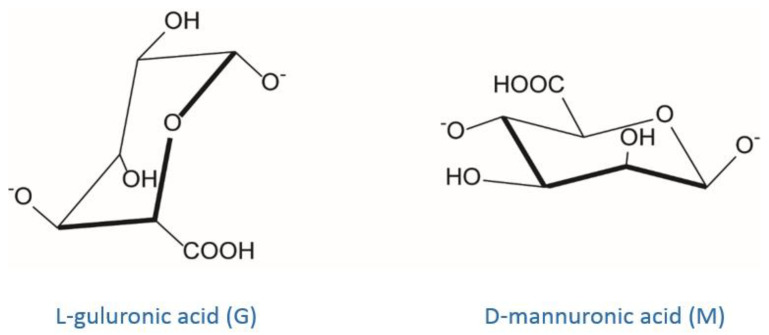
Chemical structure of alginate monomers: l-guluronic acid and d-mannuronic acid.

Alginate is certainly the most well-known and studied example of polysaccharide that can be cross-linked via a ionotropic mechanism [118]. It is a linear polysaccharide copolymer consisting of α-l-guluronic acid (**G**) and β-d-mannuronic acid (**M**) repeating units forming regions of **M**- and **G**-blocks and alternating structure (MG-blocks) [65,119,120] (Figure 7).

**Figure 8:**
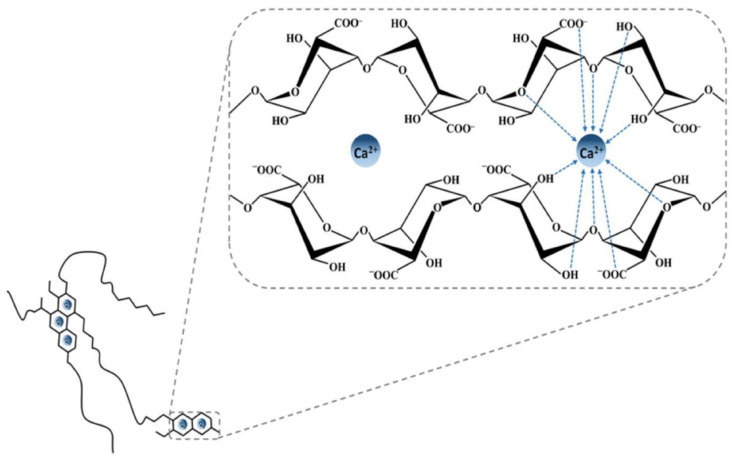
Egg-box model representing the interactions between alginate **G**-blocks and calcium ions. Reprinted from [113]. Copyright (2019) Martău, Mihai, Vodnar.

Alginate can be obtained from different brown seaweed species or from certain bacterial strains (e.g., *Pseudomonas aeruginosa*) [121]. The alginate source defines the **G**-to-**M** ratio and the molecular weight of the polysaccharide leading to significant differences in the physicochemical and mechanical properties of the resulting gels [119]. Commercially, alginates are available as alginic acid or in the form of sodium, potassium, or ammonium salts. Generally, most of divalent cations (Ca^2+^, Sr^2+^, Cd^2+^, Co^2+^, Cu^2+^, Mn^2+^, Ni^2+^, Pb^2+^ and Zn^2+^) and some trivalent cations (Fe^3+^, Cr^3+^, Al^3+^, Ga^3+^, Sc^3+^ and La^3+^) can interact with **G**-blocks regions of alginate in a highly cooperative manner, generating a 3D network according to the so-called “egg-box” model [122]. Alginate affinity towards such polyvalent cations is directly dependent on the amount of G-blocks present in the alginate structure [123,124]. As demonstrated in [125] by numerous competitive inhibition studies, the main involved gelation mechanism is the dimerization of **G** residues. In detail, the addition of polyvalent cations (often Ca^2+^ ions) to the alginate solution determines the binding of two **G**-blocks on opposite sides; the result is a diamond shaped hole consisting of a hydrophilic cavity that binds the cations by multicoordination using the oxygen atoms from the carboxyl functional groups. This arrangement causes the formation of a junction zone shaped like an “egg-box” (Figure 8).

Each cation binds with four **G** residues in the egg-box formation to form a 3D hydrogel network of these interconnected regions [15,126]. It has been reported that in the case of Ca^2+^, the formation of a stable junction requires eight to twenty adjacent **G** unites [65].

Generally, the greater the atomic radius of the cation, the stronger is the cross-linked polymeric matrix. In fact, as reported by [123,127,128,129], divalent ions of larger ionic radii such as Ba^2+^ and Sr^2+^ are able to produce stronger alginate gel particles than the Ca^2+^-based ones (Figure 8). By contrast, having a smaller atomic radius, Mg^2+^ is not able to cross-link alginate [112,130]. Overall, alginate affinity to cations increases in the following order: Mn^2+^ < Zn^2+^, Ni^2+^, Co^2+^ < Fe^3+^ < Ca^2+^ < Sr^2+^ < Ba^2+^ < Cd^2+^ < Cu^2+^ < Pb^2+^ [123]. For practical applications, the use of highly toxic cations such as Pb^2+^, Cu^2+^, and Cd^2+^ is limited. The use of Sr^2+^ and Ba^2+^, which are mildly toxic, has been reported in cell immobilization applications although only at low concentrations [131]. Ca^2+^ is certainly the divalent cation most used to form ionic alginate gels for its good binding affinity to alginate and lack of toxicity under normal conditions of use [15]. Although it is generally recognized that most divalent and trivalent cations are able to form alginate gels according to the pioneering “egg-box” model (valid mainly for **GG** sequences), it is important to consider that the chemical composition of such polymers and hence the **M**/**G** ratio can significantly vary [132]. This means that binding affinity with cations as well as polymeric conformation can vary too. Some binding studies have shown that Sr^2+^ is able to bind to G-blocks only, Ca^2+^ to both **G**- as well as **MG**-blocks, and Ba^2+^ to both **G**- and **M**-blocks [65,123]. In general, binding affinity with cations is lower for both **MM** and alternating **MG** blocks, requiring a rather high polyvalent ion concentration to be more efficiently complexed. Compared to **GG** blocks, both **MM** and **MG** ones present a more open geometry that make them more available for interchain aggregations along polymer (self-assembly of alginate chains) causing significant irregularities in the arrangement of uronic units in alginate chains; this happens for the gels cross-linked by relatively low ion radius cations [125]. Since in the pharmaceutical field the ionotropic gelation is commonly used to entrap an API between the polymer chains, this arrangement can affect the drug release in a controlled manner.

#### 3.5.2. Pectin Ionic Cross-Linking

**Figure 9:**
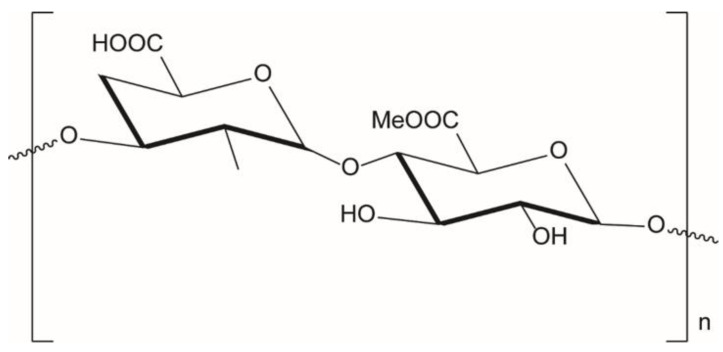
Pectin structure.

Pectin is a linear polysaccharide mainly consisting of galacturonic acid units which are connected via α-(1–4) bonds and with a certain degree of methyl esterification of carboxyl groups (Figure 9) (DE, degree of esterification) depending on the polysaccharide quality and source [133]. It is commonly extracted from apple pomace and citrus peels under slightly acidic conditions. There are currently three commercially types of pectins: (1) low methoxyl (LM) pectin, where less than 50% of galacturonic acid groups are esterified with methyl groups; (2) high methoxyl (HM) pectin, where more than 50% of existing galacturonic acid groups are esterified with methyl groups; and (3) amidated pectin (A), where acid groups are partly amidated. Gelling properties highly depend on the ratio of esterified and amidated acid groups [134].

As reported by Braccini and Perez [114], despite the structural analogy between polyguluronate and polygalacturonate chains, the egg box model valid for alginates (guluronate system) cannot be directly transposed to the pectate gels. In this case, the most favorable antiparallel associations of galacturonate chains may, at best, be considered as “shifted egg boxes”. The observed “shift” seems to lead to an efficient association with several van der Waals contacts; it reduces the original large cavity and provides two symmetrical sub-cavities of appropriate size for binding a cation and it creates an efficient periodic intermolecular hydrogen bonding network. Generally, the methyl esterification of carboxyl groups weakens the crosslinker-pectate interaction and might hamper subsequent dimer-dimer. Therefore, ionotropic gelation is more pronounced for lower-methylated pectins and at pH around 3–3.5; increasing the pH leads to deprotonation of acidic groups which prevents aggregation of chains and eventually gelation. Pectin has been also mixed with alginate to form in presence of cross-linking ions an interpenetrated network made up by heterogeneous interactions [135,136,137].

#### 3.5.3. Chitosan Ionic Cross-Linking

Among several polysaccharides amenable to ionotropic cross-linking, chitosan is another noteworthy example even if by far less investigated than negatively charged polysaccharides such as alginate and pectin [138]. Chitosan is typically obtained from the alkaline deacetylation of the highly abundant, naturally occurring polymer chitin, but other sources can directly provide it as such (e.g., yeasts) [113]. Chitosan is composed of β-1,4-linked glucosamine and *N*-acetylglucosamine residues [139]. Both the degree of acetylation (DA) and the degree of polymerisation (DP) of chitosan are crucial factors determining the structural and functional properties of this family of polymers and their resulting engineered materials [140]. Commercially available chitosans vary in their DA, usually between 5 and 20%, and in their DP or molecular weight, typically ranging between 10 and 500 kDa. Unlike chitin, chitosan is soluble in water under mild acidic conditions thanks to the protonation of its amino groups (pKa = 6.2–7) able to promote the solvation of polymer chains. In these conditions, chitosan behaves as a polycation and, consequently, can form a gel by ionic interaction in presence of multivalent anions. Tripolyphosphate (TPP) is by far the most employed cross-linker to ionically reticulate chitosan due to its high net negative charges (ranging from one to five depending on pH) per monomeric unit and nontoxicity [116,141,142]. TPP has been widely exploited in pharmaceutical field to obtain chitosan microparticles [143,144,145], nanoparticles [143,146] and nano/micro-gels [147,148] intended for controlled drug delivery [149].

### 3.6. Different Approaches to Hydrogel Formation by Ionotropic Cross-Linking

Hydrogels can be generated using ionotropic gelation technique by three main methods that differ in the way crosslinking ions are introduced to the polymer, realizing the so-called external, internal or inverse gelation [18,150].

In the external gelation or diffusion controlled method, polysaccharide solution is added dropwise into the gelling bath. The hydrogel matrix is formed through the diffusion of the cross-linking agents from the external continuous phase into the inner structure of polymeric droplets [151]. As expected, at the outermost layer of the hydrogel, gelling kinetics is rapid and gel formation is instantaneous. Then, counter-ions start to diffuse towards the center of the particle creating an inhomogeneous gelation profile in which the interaction between ions and polymer functional groups is maximum at the surface and zero at the core [152,153].

The internal gelation also called in situ gelation is an approach widely used to produce calcium alginate particles [154]. In this case, an insoluble calcium salt (e.g., CaCO_3_ and CaSO_4_) is mixed with the polysaccharide solution, and the obtained mixture is then extruded into an acidic gelling bath [155,156,157]. The acidic environment increases the solubility of calcium salt, allowing its release that leads to the formation of the polysaccharide gel network. This mechanism guarantees a controlled and homogeneous alginate exposure to cations and hence a uniform gel network formation [120]. Despite the good homogeneity, the internal cross-linked matrices results less dense, with larger pore sizes and thus more permeable than those obtained for external gelation, with lower encapsulation efficiencies and faster release rates [157,158]. This happens because matrix permeability is affected by competition between Ca^2+^ and H^+^ ions due to the acid added. It seems that while the acid in the gelling bath liberates Ca^2+^ from the insoluble salt, it also competes with Ca^2+^ for interaction with the alginate/polymer. This drawback can be overcome by manipulating the pH of the medium and the amount of calcium salt employed [150].

Another approach is the inverse gelation, based on the dripping of the medium containing the cross-linking agents into the polysaccharide solution. This method is usually applied to emulsions for producing alginate microcapsules with an oily content and soft shell [5,159,160]. In comparison to other cross-linking methods, it exploits low amounts of biopolymer leading to the formation of a soft particle shell. Clearly, as highlighted by Martins at al. [161] in a recent published paper, the properties of the obtained microcapsules (e.g., mechanical resistance and release of bioactive substances) can vary based on the type of emulsion used (W/O or O/W) for the inverse gelation

## 4. Influence of Drying Process on Gel Particle Characteristics

**Figure 10:**
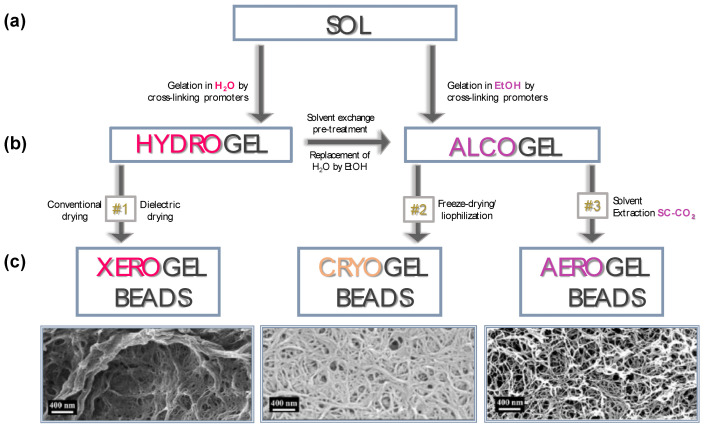
Pathway for the production of xerogels, cryogels and aerogels from polysaccharide based hydrogels via several steps including: (**a**) gelation (SOL→GEL), (**b**) solvent exchange pre-treatment, if required (e.g., the replacement of the water contained in the pores of the hydrogel with a suitable organic solvent), (**c**) drying final step with a specific technology. The images of xerogel, cryogel and aerogel beads were here reprinted (with some modifications) from [176] with permission from Elsevier. Copyright (2016).

As discussed above, polysaccharide-based hydrogel particles can be used in the hydrated form for different applications [119,162,163,164,165,166]. However, to avoid their chemical or microbiological degradation a drying step is often required [167,168]. Hydrogel particles can be dried using several techniques such as conventional, dielectric, freeze or supercritical drying, each one with a significant impact on the physicochemical and textural properties of the final dried beads [169]. Phenomena such as modifications of the highly interconnected hydrogel network, solute migration, polymorphism, damages by overheating and many other effects can occur [170]. Therefore, the choice of the drying method represents one of the critical step on the pathway to the production of PbHPs. As illustrated in Figure 10, each drying process leads to polymeric material with different inner structure; conventional or dielectric methods allow to obtain “xerogels” whereas freeze and supercritical drying generally allow to achieve “cryogels” and “aerogels”, respectively [171,172,173,174,175].

Each drying method presents specific advantages and disadvantages (Table 1), and the choice must be done based on the desired performances for the final product, system cost-effectiveness and realizable scale-up.

In the following paragraphs, production and properties of xerogel, cryogel and aerogel particles are discussed.

### 4.1. Conventional and Dielectric Drying to Produce Xerogels

Conventional drying (e.g., using ambient air and an oven) as well as dielectric treatments generally cause the collapse of the nanoporous structure of the parent hydrogel due to the high capillary pressure gradient established during the solvent removal. The collapsing of the polymer structure produces a massive volume shrinkage leading to the formation of a highly aggregated and densely packed material without pores; the formed compact structure is known as “xerogel”. However, as discussed in a large number of papers [175,177,178,179], porous texture of the dried material can be tuned by a proper selection of solvent, temperature, carrier gas used during evaporation and, specifically for microwave heating, the irradiating regimen. These parameters alone or in combination can allow to obtain either microporous, micro-mesoporous or micro-macroporous textures of the dried beads. For instance, as reported by [176] the porous structure of cellulose-based wet gels may resist to collapse to a certain limit when alcohol, having low surface tension and low vapor pressure, is used as organic solvent. In addition, an interesting study of [180] showed that degree of substitution (DS) of cellulose can play an important role for the production, via ambient drying, of low density, open porous and hydrophobic cellulose material defined “Xerocellulose”. During this research, tritylcellulose with different DS was synthesized in homogeneous conditions and then subjected to dissolution-coagulation-drying producing “Xerocellulose”. Results showed that, depending on DS, the chemical modification leads to the development of unusual microstructure due to the different manner of self-assembly of cellulose molecules and lack of hydrogen bonding.

Xerogel porosity may be specifically tuned, as demonstrated by studies of our research group aimed to verify the feasibility of the tandem technique Prilling/Microwave assisted drying for the production of alginate-based beads loaded with non-steroidal anti-inflammatory drugs (NSAIDs) [181,182]. Microwaves at different regimens of irradiation affected matrix porosity, solid state of the loaded drug (i.e., ketoprofen or piroxicam) and drug-polymer interaction, leading to beads with significant differences in drug release profiles. Interestingly, high MW irradiation level led to dried beads with highly porous and swellable inner matrix able to rapidly release the encapsulated drug in the simulated gastrointestinal fluids. By contrast, low MW irradiation levels produced beads with few pores in the inner matrix acting as NSAID delayed delivery systems.

### 4.2. Freeze-Drying to Produce Cryogels

During the freeze drying, the liquid entrapped into the hydrogel body is frozen and sublimed under regulated vacuum [118,183] reducing the volume shrinkage of the beads to 40%–50%. Unless special precautions are often taken to prevent the growth of ice crystals, freezing may destroy the pore structure and damage the nanostructured gel matrix as freezing always implies the growth of crystals [184]. The increase of the solvent volume upon crystallization induces the formation of a dendritic network of the crystalline solvent phase. The dendrites are, depending on the cooling rate, typically in the range of few up to a few tens of micrometers’ size; they push the walls of the network at the crystal boundaries destroying the morphology/inner structure [185,186,187,188,189]. The resulting material is open porous product with a pore size in the range of several micrometers termed “cryogels”.

In certain cases, modifying the freeze-drying conditions it is possible to modulate the ultrastructure of the porous matrix, moving from nanofibrillar to sheet-like skeletons with hierarchical micro- and nanoscale morphology. For instance, by increasing the cooling rate of the hydrogel precursors with e.g., liquid propane, or by using the spraying freeze drying approach, it is possible to avoid the macroporous 2D-sheet morphology of cellulose cryogels, producing aerogel-like dried systems with intermediate textural properties (BET-specific surface areas of 70–100 m^2^/g) [190]. Interestingly, the freeze drying of alcogels from resorcinol-formaldehyde using t-butanol as solvent resulted in a significant improvement of the mesoporosity of the resulting dried gels if compared to the freeze drying of hydrogel counterparts [187].

### 4.3. Supercritical Assisted Drying to Produce Aerogels

Supercritical drying is the best method to preserve the porous texture and structural properties of the wet gel network in a dry form without cracks as well as without substantial volume reduction or packed network structure due to the intrinsic absence of surface tension in the pores of the gel. Supercritical drying produces nanostructured materials with low-density (typically <0.2 g cm^−3^), high-porosity (>96% *v*/*v*) in the mesoporous range, with full pore interconnectivity and large surface area (>250 m^2^/g), commonly called “aerogels”. Aerogels have been designed in a several morphologies (e.g., cylinders, beads, microparticles) and configurations (e.g., only core, core-shell, coated particles) with an attractive processing versatility [191,192,193,194].

During supercritical drying, the organic solvent in the hydrogel pores (deriving from the pre-treatment named solvent exchange procedure, see Figure 10), is removed under supercritical conditions. As well known, a fluid reaches its supercritical state when it is compressed and heated above its critical point. Supercritical fluids have liquid-like densities and gas-like viscosities [195]. Supercritical carbon dioxide is the most commonly used fluid for supercritical drying due to its mild critical point conditions (304 K, 7.4 MPa), nontoxicity (it is considered to be Generally Recognized as Safe or GRAS), environmental friendliness, widely availability and cheapness/cost-effectiveness [191,196,197]. Generally, prior to the supercritical drying, the solvent exchange (that is the replacement of the water contained in the pores of the hydrogel with a suitable organic solvent) is needed due to the low affinity of water to supercritical carbon dioxide (SC-CO_2_) [196,198]. The presence of even small amounts of water in the pores of the wet gel can cause a dramatic change in the initially highly porous polysaccharide network upon supercritical drying. The usual approach in SC-CO_2_ drying procedure is the displacement of the water using a solvent with high solubility in CO_2_, commonly alcohol or acetone [191] and then the immersion of the gel in SC-CO_2_. The extraction time depends mainly on the thickness of the gel samples. Therefore, it can be still reduced from the several hours needed for thick monoliths to only few minutes for polysaccharide particles of millimeter size.

## 5. Case-Studies: Polysaccharide-Based Hydrogel Particles Produced by Prilling/Ionotropic Gelation and Their Application as Drug Delivery Systems (DDS)

Polysaccharide-based hydrogel particles can be used as drug carriers, which application in pharmaceutics depends on the characteristics of the polysaccharides and the drugs, the particle configuration, as well as on the inner structure, namely xerogels, cryogels and aerogels. Table 2 summarizes the different kinds of PbHPs produced via prilling/ionotropic gelation, particle configuration (only core or core-shell), their main physico-chemical and technological properties, and the potential pharmaceutical applications.

As shown in Table 2, hydrogels can be obtained as simple monolayered (only core) or multi-layered (core-shell) systems, in the hydrated or in the dried form. Based on the specific drying treatment, hydrogel beads can be transformed into xerogel, cryogel or aerogel form. The choice of a specific polysaccharide and particle system must be driven by the final desired application and performance requirements. Prior to any process design, it is necessary to study physico-chemical characteristics of the polymeric materials, like viscosities, densities, gelation and, physico-chemical and biopharmaceutical properties of the carried drug.

### 5.1. Design of PbHPs in Form of Xerogels

As reported in Table 2, successful outcomes have been achieved through a formulation design based on prilling in tandem with conventional drying that provide efficient drug delivery of both steroidal (e.g., prednisolone and betamethasone) and non-steroidal (e.g., ketoprofen, ketoprofen lysine salt and piroxicam) anti-inflammatory drugs, targeting chronic inflammation and early morning pathologies.

**Figure 11:**
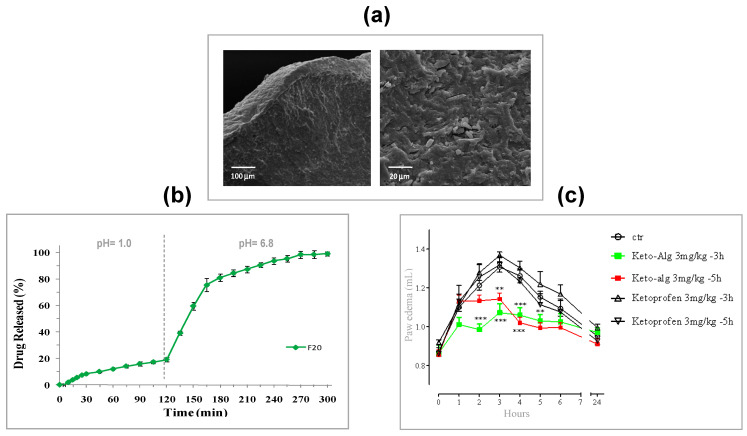
Main in vitro and in vivo results obtained for Zn-alginate-based xerogel beads loaded with ketoprofen: (**a**) SEM microphotographs showing the compact inner matrix; (**b**) relese profile performed by USP Apparatus 4 and, (**c**) edema volume reduction in rats (** *p*-value ≤ 0.01, *** *p*-value ≤ 0.001 compared with control). These images were here reprinted (with some modifications) from [202] with permission from Elsevier. Copyright (2015).

The accurate selection of biopolymer, the opportune set-up of process parameters and gelling conditions allowed to produce interesting delivery systems in the xerogel form with controlled drug release both for low soluble and highly soluble NSAIDs. Zn^2+^ as external cross-linking agent for alginate/ketoprofen (K) solutions gave PbHPs with good technological properties such as drug loading, particle size, morphology, hardness of cross-linked matrix [202]. In vitro and in vivo release behavior resulted to be strongly influenced by the amount of NSAID loaded inside the polymer; the loading of high amount of drug into feed solutions promotes, during the gelling phase, the formation of a compact gel polymeric network via intermolecular interactions, as hydrophobic or hydrogen bonding, which stabilize the well-known alginate “egg-box” structure. This phenomenon leads to tough polymer beads (Figure 11a), reducing the leaching of the drug from the drops into the gelling medium. Accordingly, the formulation obtained with the highest drug content (F20, K/alginate ratio 1:5) showed the highest entrapment of the drug within the matrix (encapsulation efficiency, 53%) and a delayed release of the drug in simulated intestinal fluid (see Figure 11b). This in vitro release pattern was clearly reflected in the in vivo prolonged anti-inflammatory effect evaluated using a modified carrageenan-induced acute edema assay in rat paw. F20, administered 3 h before edema induction, showed a significant anti-inflammatory activity, reducing maximum paw volume in response to carrageenan injection, whereas no response was observed for pure ketoprofen (see Figure 11c).

Zn^2+^ as external cross-linking agent for pectin solutions was able to produce PbHPs containing ketoprofen lysine salt (KL), a highly soluble NSAID [204]. In this case, the best results were obtained using amidated low methoxyl pectin (esterification degree 24% and amidation degree 23%) producing beads with good morphological properties and size, high drug content and encapsulation efficiency (93.5%), and interesting KL sustained release profiles.

### 5.2. Investigation on the Effect of Different Cations on Gelation Process

Many researchers evaluated the influence of different divalent cations on PbHP properties. For instance, Chan et al. [210] studied the ability of calcium chloride and zinc sulphate to cross-link alginate microspheres prepared by emulsification.

**Figure 12:**
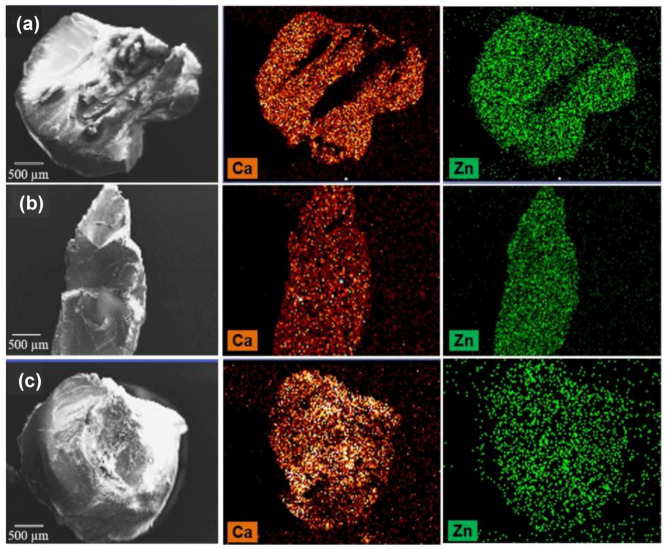
SEM and SEM-EDS microphotographs of cryofractured blank xerogel beads produced with different Ca^2+^/Zn^2+^ ratios: (**a**) 1:1, (**b**) 1:4 and (**c**) 4:1. Reprinted from [201] with permission from Elsevier. Copyright (2017).

In this study, the aqueous phase, consisting of 2.5% *w*/*w* sodium alginate and 1% *w*/*w* sulphaguanidine was dispersed in isooctane with the aid of surfactants and a mechanical stirrer. The fine globules of sodium alginate produced were gelified by addition of calcium chloride and zinc sulphate, alone or in combination. The microspheres formed were collected by filtration, washed and oven dried at 40 °C. The results of characterization studies showed that the simultaneous use of these two salts led to different particle morphology and slower drug release compared to particles cross-linked by the calcium salt alone. These effects were attributed to a greater extent of interaction between zinc cations and the alginate molecules able to produce a less permeable alginate matrix. Cerciello et al. also investigated the specific effect of these two divalent cations, i.e., Ca^2+^ and Zn^2+^, used alone or blended in different ratios (Ca^2+^:Zn^2+^, ratio 1:1, 1:4 or 4:1), on the properties of alginate beads obtained via prilling/external gelation [201]. The synergistic effect of the two cations, when used in the gelling bath in the ratio Ca^2+^:Zn^2+^ 1:4; positively affected particle morphology, size, inner structure, ability to encapsulate the model drug (SAID, prednisolone, P) and to control its release from the polymer matrix. Figure 12 shows SEM and SEM-EDS microphotographs of cryofractured blank beads obtained using the different ratios Ca^2+^/Zn^2+^ in the gelling solution. As showed, formulations gelified using Ca^2+^:Zn^2+^ in the ratios 1:1 and 4:1 exhibited an internal structure enriched in Ca^2+^, due to the higher diffusivity of this cation, compared to Zn^2+^. Only with a ratio Ca^2+^/Zn^2+^ 1:4 was possible to observe an equilibrium between the two cations quantities into the polymeric matrix. This specific ratio, in fact, exploited the Ca^2+^ ability to establish quicker electrostatic interactions with **G** groups of alginate and the Zn^2+^ ability to establish covalent-like bonds with both **M** and **G** blocks of alginate. Drug release profiles clearly reflected the advantages deriving from the simultaneous use of both cations. Their proper mixing allowed to produce a polymeric matrix tougher and more resistant compared to those obtained with a single cation (zinc or calcium) and with an interesting P prolonged release.

### 5.3. Prilling to Obtain Floating PbHPs

A significant number of studies was conducted to develop floating PbHPs, mainly alginate based particles, using gas-forming agents such as CaCO_3_ or NaHCO_3_. In the most of them, calcium has been used as external cross-linker for the gelation phase [157,211,212]. More recently, an important milestone was achieved with the simultaneous use of two different divalent cations to produce floating and prolonged release alginate PbHPs for the oral administration of prednisolone, P [207]. Critical parameters were established: prilling/ionotropic gelation was used as microencapsulation technique, zinc acetate in the gelling solution as the alginate external crosslinker, and calcium carbonate in the feed acting as the internal crosslinking agent able to generate gas when in contact with the acidic zinc acetate solution. The double gelation process (internal- and external) promoted by Ca^2+^ and Zn^2+^ ions gave alginate beads with extremely high encapsulation efficiency values (up to 94%) and a very porous inner matrix conferring buoyancy in vitro in simulated gastric fluid up to 5 h. Particularly, the best formulation F4 (P/Alginate ratio 1:5; Alg/CaCO_3_ ratio 1:0.50) was able to control the drug release in acidic medium for the entire time corresponding to the floating period. Although porous, the tougher matrix obtained thanks to the double gelation process is able to reduce swelling and erosion processes in simulated gastric fluid (SGF). F4 was also able to prolong the in vivo anti-inflammatory effect up to 15 h compared with raw prednisolone. Therefore, this alginate-based system has been proposed as a new technological platform able to extend the anti-inflammatory efficacy of SAID such as prednisolone (characterized by high efficacy and high tolerability, but short half-life) for many hours and successfully treat patient suffering from chronic inflammatory diseases, also reducing the frequency of the oral administration.

An interesting production innovation was obtained designing floating PbHPs with controlled release properties without using any gas-generating agent. Our research group designed a hollow multipolymer matrix made up of alginate, ALM-pectin and HPMC. Results showed that particle shape and sphericity can be correlated to nozzle viscosity of the feed solutions; the higher the nozzle viscosity, the slower the break-up of the polymeric laminar-jet and, thus, droplet formation. The high entanglement existing between the chains of three different polymers makes the polymeric jet highly cohesive (viscoelastic stresses dominate) delaying drops detachment from the nozzle. At the lowest feed concentration (4.75 *w*/*w*), corresponding to a nozzle viscosity of 24.4 mPa·s, polymer chains are relaxed and surface tension dominates, allowing the formation of droplets that, falling in the gelation bath, give rise to spherical particles. Optimized formulation F4 (Drug/Polymers ratio 1:15; Pol_1_/Pol_2_/Pol_3_ ratio 1.25:3:0.5) showed beads spherical in shape with a sphericity coefficient mean value of 0.94 and a mean diameter around 2200 μm. This formulation acts as a floating-system able to release the encapsulated model drug (piroxicam, PRX) in a controlled and delayed manner.

**Figure 13:**
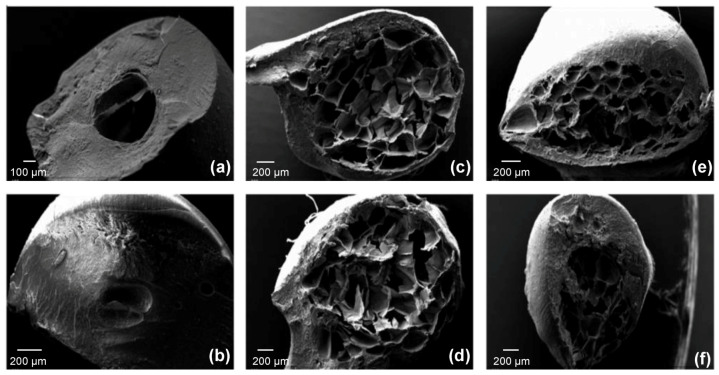
SEM microphotographs showing the hollow inner matrix of polysaccharide-based floating beads produced with different Pol_1_/Pol_2_/Pol_3_ ratios: (**a**,**b**) 1.75:4:1, (**c**,**d**) 1.75:3:0.5 and (**e**,**f**) 1.25:3:0.5. Reprinted from [206] with permission from Elsevier. Copyright (2018).

**Figure 14:**
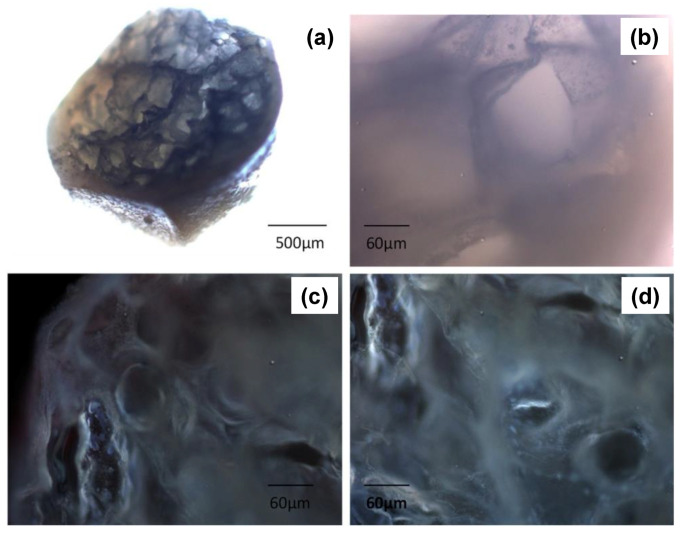
Microphotographs obtained using bright-field (**a**,**b**) and fluorescent microscopy (**c**,**d**) showing the presence of pores and air bubbles entrapped within the gel matrix of hydrated beads, able to confer their floating ability in SGF. Reprinted from [206] with permission from Elsevier. Copyright (2018).

Floating properties of F4 are due both to the swelling of the hydrocolloid particles and to hollow inner structure (Figure 13). The hydration of the hydrocolloid particle surface in SGF results in an increased bulk volume and, at the same time, the presence of internal pores make beads able to entrap air. As a result beads had bulk density <1, and, therefore, remained buoyant on the acidic medium. In addition, the inner cross-linked multi-polysaccharide matrix acts as reservoir for slow and sustained PRX. Drug release was controlled by a diffusion mechanism process through the swollen polymers gel layer, as shown by in vitro release assay. Figure 14 shows the presence of pores and air bubbles entrapped within the gel barrier after 60 min of floating in acidic medium.

As expected by morphology and results from in vitro assays, a promising application of floating PbHPs is the treatment of chronic inflammatory-diseases in elderly patients needing a rapid onset of drug action followed by a maintenance dose. In this regard, the in vivo anti-inflammatory activity of this type of new floating PbHPs, evaluated using the modified protocol of carrageenan-induced acute edema in rat paw previously developed [202], showed an incredible extension, up to 48 h, of the anti-inflammatory effect compared to standard PRX, as effect of both floating and sustaining release abilities.

### 5.4. Core-Shell PbHPs

Generally, the production of core-shell PbHPs through a generic dripping device can be easily conducted exploiting coacervation. The major driving force for the coacervation method is electrostatic attraction between cationic and anionic water-soluble polysaccharides. The resultant “coacervate” generally forms the particle shell. The power of the interaction between the polysaccharides and the nature of the complex is affected by many factors such as pH, concentration, ionic strength, biopolymer type, and the ratio of biopolymers [213]. With this method several core-shell PbHPs have been produced [214,215,216]. For instance, alginate and chitosan can be used together because of their opposite charges to form alginate particles coated with chitosan. The electrostatic interaction of carboxylic groups of alginate with the amine groups of chitosan results in the formation of a membrane surrounding the surface of the core particles and reduces their porosity [47,217,218]. Ren et al. [219] exploited this method to prepare alginate-chitosan microcapsules for protein delivery via oral route. This study confirmed that such systems are pH sensitive; in acidic solution, due to the ionic bond between the chitosan and alginate as well as the physical barrier provided by the interphasic membrane itself, microcapsules maintained integrity well, and effectively prevented the direct exposure of protein to the gastric fluid. In fact, less than 10% of protein (i.e., IgG) was released in SGF and 80% of its activity was preserved; during the first hour of permanence into simulated intestinal fluid, a burst release of IgG was observed.

**Figure 15:**
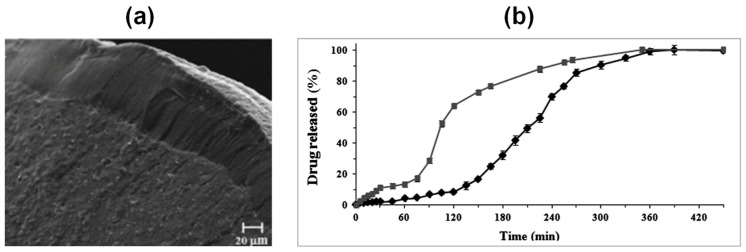
Main in vitro results obtained for core-shell (Pectin/Alginate) beads loaded with piroxicam: (**a**) SEM microphotographs showing the complete and homogeneous alginate shell surrounding the pectin core; (**b**) Release profiles of mono-layered “only core” (Pectin) beads (-■-) and bi-layered “core-shell” (Pectin/Alginate) beads (-♦-), performed in simulated intestinal fluid by using USP Apparatus 2. Reprinted from [43] with permission from Elsevier. Copyright (2014).

In general, to increase the performances of such particle systems and make more efficient the delivery of macromolecules as well as drugs throughout the GIT, the manufacturing phase should provide a major control on shell formation process. To this regard, an important achievement was the design and the development of multiparticulate beads in core-shell configuration using (a) prilling apparatus in its basic configuration followed by an enteric coating process [32,205] and (b) prilling apparatus in coaxial configuration [31,43]. This latter is certainly the most innovative approach; it employs multiple concentric nozzles to produce a smooth coaxial jet comprising polymer annular shell and core material, which are broken up by acoustic excitation into uniform core-shell droplets and gelled into a cross-linking solution. PbHPs in core-shell configuration consisting of zinc-ALM pectinate as core and zinc-alginate as shell were produced. Both NSAID (piroxicam, PRX) and SAID (betamethasone, B) were respectively loaded as model drug within the pectin core. The aim was to combine the pH dependent solubility (gastro-resistance) of zinc alginate and the colon targeted selectivity of zinc-ALM pectinate [220,221] in a unique system to obtain an enteric carrier targeting colon. The most critical process parameter to obtain uniform double-layered particles was identified in the ratio between the nozzle viscosity of the inner and outer polymer solutions. In fact, beads with homogeneous layered, good spherical shape and smooth particle surface were obtained when this ratio was above 6. Moreover, optimization of other process parameters such as selection of the cross-linker, pH of the gelling solution as well as cross-linking time was necessary to obtain well-formed and homogeneously coated microcapsules (see Figure 15, panel a) with strong drug/polymer core showing a tailored control of drug release in the gastrointestinal tract (see Figure 15, panel b).

### 5.5. Design of PbHPs in Form of Aerogels

The application of biopolymer aerogels as drug delivery systems has gained increased interest during the last decade since these structures have large surface area and accessible pores allowing for, e.g., high drug loadings. Examples of oral, mucosal, and most recently pulmonary drug delivery routes have been highly discussed in the literature. Furthermore, thanks to high pore volume and swelling ability both pristine and drug-loaded polysaccharide aerogel particles have been suggested as superabsorbent and for wound healing applications [44,222]. Being largely mesoporous solids, aerogels can accommodate drugs in the amorphous state suppressing re-crystallization [223]. This feature along with the high specific surface area and rapid pore collapse upon contact with liquid media gives rise to unusually fast drug release.

**Figure 16:**
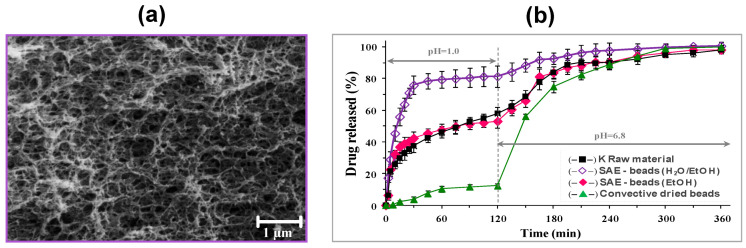
Main in vitro results obtained for alginate-based aerogel beads loaded with ketoprofen: (**a**) SEM microphotographs showing the inner nanoporous structure; (**b**) Release profiles of beads dried by both supercritical-CO_2_ and conventional drying, performed in simulated gastro-intestinal fluids by using USP Apparatus 2. Reprinted from [208] with permission from Elsevier. Copyright (2012).

Reverchon et al. [208,209] produced alginate-based aerogels as carriers for the fast delivery of slightly soluble NSAIDs in the upper gastrointestinal tract by prilling of drug/alginate feed solutions followed by cross-linking in ethanol or aqueous CaCl_2_ solutions, water replacement and, supercritical-CO_2_-assisted drying. The selected techniques allowed to successfully produce spherical aerogels (sphericity coefficient 0.97–0.99) in narrow size distribution with reduced particle shrinkage and smooth surface (surface roughness 1.10–1.13); the internal porous texture of the parent hydrogels was preserved and appeared as a network of nanopores with diameters around 200 nm (see Figure 16a).

Recently, we verified the influence of the alginate molecular weight, the solvent used in the gelation solution on porosity, textural properties and stability of the alginate aerogel beads produced via prilling/ionotropic gelation/SC-CO_2_ drying route [169]. Gelation in ethanolic media promoted the formation of aerogels with higher textural properties compared to the aerogels derived from particle crosslinked in aqueous media. As expected, the textural properties of aerogels were far higher than those obtained from cryogels and xerogels obtained by freeze-drying and oven drying, respectively. This study also highlighted that the use of medium molecular weight alginate led to aerogels with reduced shrinkage and enhanced porosity. By contrast, the use of high molecular weight alginate promoted the formation of aerogels with higher surface area. Finally, stability studies showed non-significant variations in aerogels weight and specific surface area after 3 months of storage, especially, in the case of aerogels produced with medium molecular weight alginate. Overall, this study allowed to highlight the suitability of such materials for wound dressing applications. In fact, thanks to their high surface area, aerogels can rapidly absorb the exudate once applied on a wound and at the same time, they can promote a controlled release of the active substance eventually embedded within the polymer network.

Several other researchers developed aerogel-based formulations for the management of chronic wounds. For instance, with this aim López-Iglesias and coworkers [224] produced vancomycin-loaded chitosan aerogel beads, starting from the simple dripping of a chitosan solution into a basic NaOH 0.1 M solution. In this sol-gel method, the gelation took place immediately after contact with the medium. After that, the productive process continues with the solvent exchange carried out using absolute EtOH, and it ends with the SC drying. The dried particles showed a fibrous structure characterized by a high porosity (>96%) and large surface area (>200 m^2^/g); they preserved their initial spherical structure, with an overall volume shrinkage of 57.0 ± 4.5% attributable to the flexibility of the polymeric chains of chitosan that are brought closer after the extraction of the solvent.

A significant number of investigations also focused on the possibility to produce layered aerogels. Veronovsky et al. [225] prepared multilayer amidated LM pectin aerogel particles via ionotropic gelation by dripping 2% Wt pectin solutions through a needle into calcium chloride solution. The obtained hydrogels were then dripped into a 1% Wt pectin solution for obtaining membranes around the core particles, and again were crosslinked in CaCl_2_ solution. Three-membrane hydrogel particles were produced by repeating the process. After solvent exchange, particles were dried with supercritical CO_2_. To verify the ability of such materials to act as carriers for drug delivery, two model drugs that is theophylline and nicotinic acid were loaded within the core. The selected operative conditions allowed obtaining multilayer pectin aerogel particles with diameter of 8.0 and 9.8 mm, depending on the source of pectin (apple and citrus, respectively). Specific surface area varied from 469 to 593 m^2^/g based on pectin source and its concentration. The release of both loaded drugs turned out to be controlled by swelling and dissolution of pectin matrix. Aerogels from citrus pectin showed more controlled release behavior than those from apple. Moreover, core-shell aerogels have been designed to delivery antibiotics. De Cicco et al. [44] combined amidated LM pectin with alginate to produce core-shell aerogels loaded with doxycycline hyclate, by means of prilling technique in coaxial configuration. Ionotropic gelation was conducted via diffusion method (external gelation) in an ethanolic CaCl_2_ solution. The obtained aerogels showed spherical shape, smooth surface and an apparent density of around 0.3 g/cm^3^.

### 5.6. Design of Experiments (DoE) and Artificial Intelligence (AI) for the Productionof PbHP by Inverse Gelation Technique

Recently, our research group exploited the possibility to apply the Design of Experiment for the development of wet alginate soft-capsules with a hydrophilic core through inverse gelation [199]. Soft-capsules, designed for topical administration, were produced through a vibrating nozzle device, dripping a thickened calcium chloride solution into an alginate bath. The experimental design applied revealed the effect of several critical process parameters such as the solution’s composition, frequency and flow-rate onto critical quality attributes of the produced soft-capsules as e.g., drug content, encapsulation efficiency, size, shape and mechanical strength. The overall knowledge gained through the DoE exercise showed that the inverse gelation process was capable of producing PbHPs as soft-capsules with the desired attributes, resistant enough to allow handling during storage, but also very easy to break when applied onto the skin.

**Figure 17:**
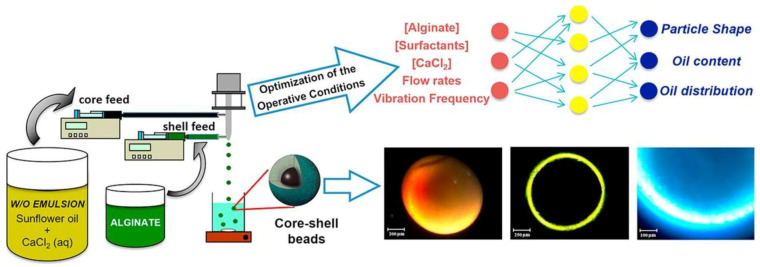
Schematic illustration of the method employed for the production of core-shell microparticles by inverse gelation optimized with artificial intelligent tools. Reprinted from [200] with permission from Elsevier. Copyright (2018).

Process optimization may be gained by integration of several methods into the process of the formulation design of PbHPs. Artificial intelligence (AI) is one of the possibilities to predict the optimal process conditions, reproducibility and great precision and accuracy in data analysis. In the last years, we proposed inverse gelation for the production of PbHPs as wet core-shell microcapsules using AI tools for the process optimization [200]. A w/o emulsion containing aqueous calcium chloride solution in sunflower oil pumped through the inner nozzle of a prilling encapsulator apparatus gave the core while an aqueous alginate solution, coming out from the annular nozzle, produced the particle shell (see Figure 17). The numerous operative conditions such as w/o constituents, polymer concentrations, flow rates and frequency of vibration were optimized by two commercial software, FormRules^®^ and INForm^®^, which implement neurofuzzy logic and artificial neural networks together with genetic algorithms, respectively. The optimized parameters by AI tools allowed to manufacturing of spherical core-shell beads (sphericity coefficient about 0.98) with diameter about 1.1 mm and a narrow size distribution containing the oily droplet wrapped by a thin and regular alginate layer (about 95 μm). This technique certainly represents an innovative approach to produce PbHPs in form of core-shell particles with different hardness loaded with oil or drugs dispersed into lipids.

## 6. PbHPs: Safety by Design and Clinical Translations

Despite the largely evolving knowledge and techniques for the development of PbHPs as controlled DDS able to improve biopharmaceutical properties of various bioactive molecules (e.g., small molecules, protein, oligonucleotides), their clinical study remains very limited [2]. As it always happens, opportunities are accompanied by challenges, and for new process technologies in pharmaceutical field, the main issue is the insecurity concerning their safe implementation. The main difficulty to translate such systems into clinical studies is due to a general lack of:

(1) understanding how properties of such materials influence the adsorption of bioactive molecules and their effect on cellular reactions,
(2) standardized methods assessing material characteristics as well as biological reactions,

both aspects falling in the field of safety. In particular, it is important to consider that natural-based polysaccharides are not a single discrete chemical system, as they vary in number and distribution of repeating building blocks along the backbone [2]. Since polymer molecular weight and composition can significantly vary, their main physicochemical properties such as solubility, chain flexibility, intra- and intermolecular forces, carrier size/shape, loading capacity, surface charge, and degradation profile can vary, and consequently also the in vivo performances. These aspects must be taken into account by regulatory authorities during control phases for a successful translation of PbHPs from bench to bedside.

In this contest, the safety-by-design (SbD) approach acquires a significant relevance. In general, SbD concepts foresee the risk identification and reduction as well as uncertainties regarding human health and environmental safety during the early stages of product development, by altering its design and by ensuring safety along its lifecycle. The SbD concept is therefore different from conventional risk assessment approaches, which only consider safety when the product is already fully developed. As well known, while the concept of quality-by-design (QbD) is widely used by pharmaceutical industry and its implementation is foreseen by the pharmaceutical development guidelines [226,227], that of SbD is new, and it is not yet included in ICH, EMA, or FDA guidelines. This means that even if safety is taken into account during the pharmaceutical development, there is still no systematic SbD approach in place, yet. As the QbD requires for its application the definition of the critical quality attributes (CQA) that will lead to the achievement of a product with proven effectiveness, in the same way the SbD has to establish CQA leading to a product with low safety concerns. Few papers focus on this intriguing and complex topic involving multidisciplinary knowledge (i.e., life science, clinical medicine, material science, chemistry, and engineering) as well as active collaboration among regulatory authorities, pharmaceutical companies, academics, and governments.

**Figure 18:**
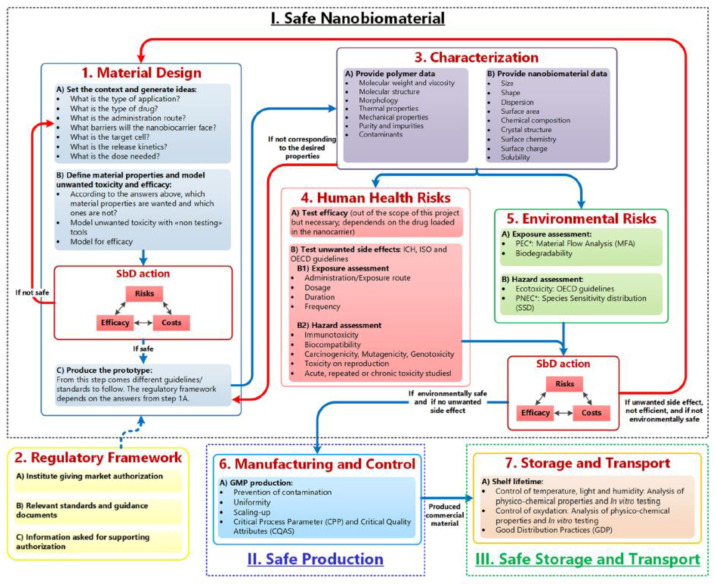
Safety-by-Design approach. Blue arrows correspond to the flow of polymeric nanobiomaterials as drug delivery systems from design to storage and transport, red arrows are feedback loops uses whenever the nanobiomaterial product is unsafe, inefficient or has unwanted side effects, and bullet points represent the methods/tools or endpoints at each step. Reprinted from [228]. Copyright (2020) Schmutz, Borges, Jesus, Borchard, Perale, Zinn, Sips, Soeteman-Hernandez, Wick and Som.

Recently, Schmutz et al. [228] elaborated a useful methodological SbD approach (referred as GoNanoBioMat SbD approach) focusing on polymeric biomaterials widely used to prepare nanoparticles and microparticles for drug delivery. Such approach allows identifying and addressing the relevant safety aspects to face with when developing biopolymeric-based DDS during design, characterization, assessment of human health and environmental risk, manufacturing and handling. As shown in Figure 18, the pillars of such approach are the following:

(1) Material Design, Characterization, Human Health and Environmental Risks
(2) Manufacturing and control
(3) Storage and Transport.

For the Human Health Risks step, the route of administration/exposure, the dosage, the duration and frequency should be determined as safety of polymeric biomaterials depends on the route of administration/exposure and the resulting respective pharmacokinetic profile. If one final candidate has been selected, the developer of such materials should go to the Manufacturing and Control step to ensure product safety and quality. The goal of this step is to scale-up the production by applying Good Manufacturing Practices (GMPs), preventing contamination and ensuring uniformity between the batches. In this step, CQAs of nanobiomaterials must be identified as well as Critical Process Parameters. These are defined as the “process parameters that influence CQAs and therefore should be monitored or controlled to ensure the process produces the desired quality” (ICH Q8 (R2), 2009). The goal of the final step is guarantee safe storage and transport.

A recent paper by Poel and Robay [229] highlights the usefulness to “design for the responsibility for safety”, rather than directly for safety, and propose some heuristics to use in deciding how to share and distribute responsibility for safety through design; in summary, designers should think where the responsibility for safety is best situated and design technologies, accordingly. The solution to safety issues is not to be sought in transferring all responsibility to the users of a technology (or to other stakeholders) but rather in a model of responsibility shared among the various actors involved, such as operators and users. The authors state that it is better to accept indeterminacy and to use it as potential source for safety rather to design it out in an attempt to achieve absolute safety, which is unattainable; in many real-world situations, human actions should be considered not a source of risks but of safety. Reason argues that the human possibility to improvise may be crucial to react to (unexpected) risks and is, therefore a source of safety [230]. Based on these considerations, indeterminacy is not only a liability but also an asset as it opens the possibility to use the expertise and insights of the actors in the value chain to identify risks unknown during the design phase.

To date, there are still many open questions on safety and conformity assessment. From this point of view, an early and deep dialogue between experts from academic community, industry and regulatory authorities is of utmost importance to “anticipate” quality and safety requirements of PbHPs. In general, regulatory requirements as well as regulatory/scientific guidance on new technologies and nanomaterials applied in medicinal products as well as medical devices are emerging. A new regulatory framework for medical devices was recently published in Europe [231]. The new regulation contains several provisions for nanomaterials, including a definition, specific attention for safety of nanomaterials and classification rules leading to different routes for conformity assessment. For the implementation of such aspects, more guidance is needed.

## 7. Conclusions

In this review, a comprehensive description of the theoretical and practical aspects behind the production of different polysaccharide-based hydrogel particles (PbHPs) by prilling technology in tandem with several curing technique is given. PbHPs, depending on the designed characteristics, can be produced as drug delivery systems exerting a number of functions as to control drug release and targeting. Beads with modular inner structure and tailored texture (xerogel, aerogel, cryogel) and with different configurations (mono-layered “only core” or multi-layered “core-shell”) may be obtained by a proper selection of the droplet formation technique and the subsequent gelation step. However, the choice of the drying method for the hydrogel is a critical step allowing to obtain carriers with specific morphology and inner structure and hence with controlled release properties, mainly of anti-inflammatory and antibiotic drugs.

Several studies in literature highlight that prilling is a versatile technique to develop polysaccharide-based particles loaded with different drugs (i.e., NSAIDs and SAIDs), both with poor and high solubility, intended for oral or topical applications and intended as fast or prolonged/sustained drug release formulations. The added value of this technique is related to its versatility and scalability. In fact, such technique is already approved for large scale by some companies. In general, the pathway for PbHP production through prilling comprises a deep knowledge of various formulation and process parameters, starting from biopolymer concentration, type of solvent, feed solution viscosity, gelling methods and properties of cation or cations for ionotropic gelation. Moreover, drying conditions play an important role in determining the characteristics of the final material.

Despite the promising pharmaceutical applications, these are some challenges correlated to the numerous critical parameters influencing size, shape, morphology and structure (i.e., homogeneity, density, strength, flexibility, pore size, permeability) of the final material and hence the release profile of the entrapped drug and its in vivo activity. The possibility to apply the Design of Experiment for the development of PbHPs and the use of artificial intelligent (AI) tools for the process optimization may reduce the time and make more efficient the process design. Another important aspect to consider is the possibility of including safety in the design of PbHPs as useful tool to enhance the medical translation of such innovative DDS.

## Figures and Tables

**Table 1:** Main characteristics of the drying methods for polysaccharide hydrogels.

Drying Method	Particle Inner Structure	Advantages	Disadvantages
Conventional Dielectric Drying	XEROGEL	▪ Simple, rapid and cheap ▪ High shrinkage	▪ Presence of capillary forces that destroy part of the inner structure
Supercritical-Assisted Drying	AEROGEL	▪ No shrinkage of the porous inner texture	▪ High cost ▪ Need of aqueous/organic solvent exchange
Freeze-Drying	CRYOGEL	▪ Low shrinkage ▪ Higher pore Diameter	▪ High cost ▪ Need of aqueous/organic solvent exchange

**Table 2:** Overview of PbHPs (polysaccharide-based hydrogel particles) produced by prilling/inotropic crosslinking/drying methods.

Polysaccharide	Prilling Configuration	Ionic Cross-Linking Conditions	Drying Method	Type of Particle/ Inner Structure	Pharmaceutical Application	References
Alginate	Basic Apparatus	Inverse gelation Ca^2+^	None	Soft alginate capsules	Topical administration	[199]
Alginate	Coaxial system	Inverse gelation Ca^2+^	None	Hydrated core-shell beads loaded with hydrophobic substances	Microencapsulation of hydrophobic compounds into a hydrophilic matrix	[200]
Alginate	Basic Apparatus	External gelation Ca^2+^, Zn^2+^, Ca^2+^ plus Zn^2+^	Conventional	Only core Xerogels	Delayed DDS for oral administration	[23,29,201,202,203]
Alginate	Basic Apparatus	External gelation Ca^2+^	Dielectric	Only core Xerogels	Controlled DDS for oral administration	[181,182]
Pectin	Basic Apparatus	External gelation Zn^2+^	Conventional	Only core Xerogels	Delayed DDS for oral administration	[204]
Pectin	Basic Apparatus plus enteric coating ES100	External gelation Zn^2+^	Conventional	Core/shell beads Xerogels	Colon targeted DDS for oral administration	[32,205]
Pectin and Alginate	Coaxial system	External gelation Zn^2+^	Conventional	Core/shell beads Xerogels	Colon targeted DDS for oral administration	[31,43]
Alginate, Pectin and HPMC	Basic Apparatus	External gelation: Zn^2+^	Conventional	Floating Hollow Beads	Floating and sustained release DDS for oral administration	[206]
Alginate	Basic Apparatus	(a) External gelation Zn^2+^ (b) Internal gelation Ca^2+^	Conventional	Floating Hollow Beads	Floating and sustained release DDS for oral administration	[207]
Alginate	Basic Apparatus	External gelation Ca^2+^	Supercritical-CO_2_	Only core Aerogels	Immediate release DDS for oral administration	[208,209]
Alginate and Pectin	Coaxial system	External gelation Ca^2+^	Supercritical-CO_2_	Core/shell Aerogels	Topical application (Wound Healing)	[44]
Alginate	Basic Apparatus	External gelation Ca^2+^	▪ Conventional ▪ Freeze-drying ▪ Supercritical-CO_2_	▪ Xerogels ▪ Cryogels ▪ Aerogels	Controlled DDS for oral administration or topical application	[169]
